# Complete Genome Sequence of a Divergent Human Rhinovirus C Isolate from an Infant with Severe Community-Acquired Pneumonia in Colorado, USA

**DOI:** 10.1128/genomeA.01245-17

**Published:** 2017-11-30

**Authors:** Charles Langelier, Corin V. White, Marci K. Sontag, Peter M. Mourani, Joseph L. DeRisi

**Affiliations:** a Division of Infectious Diseases, Department of Medicine, University of California San Francisco, San Francisco, California, USA; b Department of Biochemistry and Biophysics, University of California San Francisco, San Francisco, California, USA; c Department of Epidemiology, Colorado School of Public Health, University of Colorado Denver Anschutz Medical Campus, Aurora, Colorado, USA; d Section of Critical Care Medicine, Department of Pediatrics, University of Colorado School of Medicine and Children’s Hospital Colorado, Aurora, Colorado, USA; e Chan-Zuckerberg Biohub, San Francisco, California, USA

## Abstract

Here, we report the genome sequence of a divergent human rhinovirus C isolate identified from an infant with a severe community-acquired respiratory infection. RNA sequencing performed on an Illumina platform identified reads aligning to human rhinovirus species, which were *de novo* assembled to produce a coding-complete genome sequence.

## GENOME ANNOUNCEMENT

Human rhinoviruses can induce a diverse spectrum of clinical outcomes, ranging from mild disease to fulminant pneumonia (1–3). The factors governing the severity of rhinoviral infection are not well understood, but species- and type-specific differences are suspected to be influential (1–3). The genomic study of rhinovirus isolates associated with distinctly severe clinical syndromes may illuminate such connections. As such, we report the coding-complete genome sequence of a divergent human rhinovirus species C isolate recovered in March 2015 from the tracheal aspirate of a 15-month-old infant with a severe community-acquired lower respiratory tract infection. The previously healthy infant developed progressively worsening cough and congestion that advanced to acute respiratory failure and septic shock. A clinical respiratory virus PCR assay returned positive for rhinovirus, but all other testing, including bacterial cultures from tracheal aspirate and blood, returned negative. Following 8 days of intensive care unit support involving mechanical ventilation, vasopressors, and empiric broad-spectrum antibiotics, he fully recovered.

Following enrollment in a research study investigating factors predisposing to ventilator-associated pneumonia (IRB 14-1530), excess tracheal aspirate from the day of intubation was collected. RNA extraction and subsequent reverse transcription were carried out followed by sequencing library construction using the Illumina Nextera library preparation kit according to previously described methods (4–6). Paired-end sequencing on an Illumina instrument generated 9.9 × 10^6^ raw reads which were parsed through a recently described pathogen detection computational pipeline that incorporates iterative filtration to remove the human genome and low-quality and low-complexity sequences (4–6). From this, 58,922 unique reads aligning to human rhinovirus species were identified. Paired-read iterative contig extension (PRICE) (7) software was subsequently employed for *de novo* assembly to generate a 7,056-bp contiguous sequence, which was found to be most phylogenetically related to human rhinovirus C isolate LZ269 (GenBank accession number JF317013). The Picornavirus Working Group has established that novel human rhinovirus C types should exhibit at least 13% nucleotide sequence divergence in the VP1 protein. This isolate is 23% divergent in VP1 and 21% divergent when compared across the entire genome (8).

### Accession number(s).

The rhinovirus C isolate described here has been deposited at DDBJ/EMBL/GenBank under the accession number MG148341.
